# The *matlockite*-type praseodymium(III) oxide bromide PrOBr

**DOI:** 10.1107/S1600536811048227

**Published:** 2011-11-23

**Authors:** Pia Talmon-Gros, Christian M. Schurz, Thomas Schleid

**Affiliations:** a Institut für Anorganische Chemie, Universität Stuttgart, Pfaffenwaldring 55, 70569 Stuttgart, Germany

## Abstract

The crystal structure of the praseodymium(III) oxide bromide, PrOBr, can be best described with layers of agglomerated square anti­prisms [PrO_4_Br_4_]^9−^. These slabs are stacked along the *c* axis and linked *via* two different secondary contacts between Pr^3+^ and Br^−^. The Pr^3+^ cations occupy the Wyckoff site 2*c* with 4*mm* symmetry and carry four O^2−^ anions as well as four primary Br^−^ anions, yielding a coordination number of 8. While the Br^−^ anions exhibit the same site symmetry as the Pr^3+^ cations, the oxide anions are located at the Wyckoff position 2*a* with site symmetry 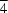 *m*2 and have four Pr^3+^ cations as neighbours, defining a tetra­hedron.

## Related literature

For prototypic PbFCl (mineral name: *matlockite*), see: Nieuwenkamp & Bijvoet (1932 ▶) and for an early powder study, see: Mayer *et al.* (1965 ▶). For other PrO*X* structures, see: Baenziger *et al.* (1950 ▶) for *X* = F, Zachariasen (1949 ▶) for *X* = Cl, and Potapova *et al.* (1977 ▶) for *X* = I. For data used for a comparison of the unit-cell dimensions, see: Shannon (1976 ▶) for ionic radii and Biltz (1934 ▶) for volume increments. For a proper classification of primary and secondary contacts, see: *MAPLE* (Hoppe, 1975 ▶) and for the bond-valence method, see: Brown (2002 ▶). For a comparison of intended synthesis attempts, see: Mattausch & Simon (1996 ▶); Lulei (1998 ▶).

## Experimental

### 

#### Crystal data

PrOBr
*M* *_r_* = 236.82
Tetragonal, 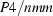

*a* = 4.0671 (3) Å
*c* = 7.4669 (5) Å
*V* = 123.51 (2) Å^3^
*Z* = 2
Mo *K*α radiation
μ = 35.52 mm^−1^
*T* = 293 K
0.11 × 0.07 × 0.02 mm

#### Data collection

Bruker–Nonius KappaCCD diffractometer
Absorption correction: numerical (*X-SHAPE*; Stoe & Cie, 1999 ▶) *T*_min_ = 0.049, *T*_max_ = 0.535
1621 measured reflections
113 independent reflections
111 reflections with *I* > 2σ(*I*)
*R*_int_ = 0.082

#### Refinement

*R*[*F* ^2^ > 2σ(*F* ^2^)] = 0.026
*wR*(*F* ^2^) = 0.059
*S* = 1.20
113 reflections
10 parameters
Δρ_max_ = 1.14 e Å^−3^
Δρ_min_ = −2.52 e Å^−3^

### 

Data collection: *COLLECT* (Nonius, 1998 ▶); cell refinement: *SCALEPACK* (Otwinowski & Minor, 1997 ▶); data reduction: *SCALEPACK* and *DENZO* (Otwinowski & Minor, 1997 ▶); program(s) used to solve structure: *SHELXS97* (Sheldrick, 2008 ▶); program(s) used to refine structure: *SHELXL97* (Sheldrick, 2008 ▶); molecular graphics: *DIAMOND* (Brandenburg, 2006 ▶); software used to prepare material for publication: *SHELXL97*.

## Supplementary Material

Crystal structure: contains datablock(s) I, global. DOI: 10.1107/S1600536811048227/fi2117sup1.cif

Structure factors: contains datablock(s) I. DOI: 10.1107/S1600536811048227/fi2117Isup2.hkl

Additional supplementary materials:  crystallographic information; 3D view; checkCIF report

## Figures and Tables

**Table 1:** Selected bond lengths (Å)

Pr—O 4 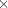	2.3496 (3)
Pr—Br 4 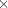	3.2457 (8)
Pr—Br	3.6083 (14)
Pr—Br	3.8586 (14)

**Fig. 1.**
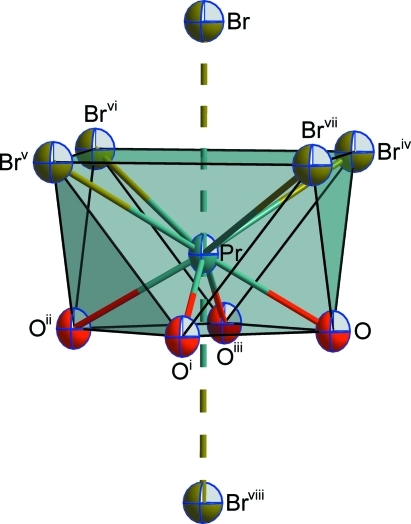
View at the square antiprism [PrO4Br4]9– with two different Br- caps in matlockite-type PrOBr. Displacement ellipsoids are drawn at 90 % probability level. Symmetry codes: (i) -x+1, -y, -z; (ii) x-1, y, z; (iii) -x+1, -y+1, -z; (iv) -x+1, -y+1, -z+1; (v) -x, -y, -z+1; (vi) -x, -y+1, -z+1; (vii) -x+1, -y, -z+1; (viii) x, y, z-1.

**Fig. 2.**
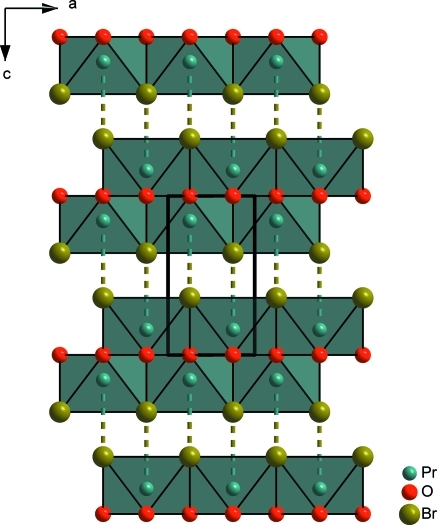
Polyhedral representation of the matlockite-type PrOBr structure (dotted lines indicate the first of the two kinds of secondary contacts between Pr3+ and Br-).

## supplementary crystallographic information

### Comment

With the exception of PrOF (Baenziger *et al.* 1950) all praseodymium(III) oxide halides of the general composition PrO*X* (*X* = Cl – I; Zachariasen 1949, Potapova *et al.* 1977) crystallize with the *matlockite*-type structure (Nieuwenkamp & Bijvoet, 1932). The tetragonal crystal structure of the here presented praseodymium(III) oxide bromide PrOBr can be best described with layers of agglomerated square antiprisms [PrO_4_Br_4_]^9–^ (*d*(Pr^3+^–O^2-^) = 234.96 (4) pm, *d*(Pr^3+^–Br^-^) = 324.57 (8) pm, *d*(Pr^3+^···Br^-^) = 360.8 (1) and 385.9 (1) pm; Figure 1). These slabs are stacked along the *c*-axis and linked via two different secondary contacts between Pr^3+^ and Br^-^ (Figure 2). According to the ionic radii (*r*_Cl_ = 180 pm, *r*_Br_ = 195 pm, *r*_I_ = 220 pm; Shannon, 1976) of the halide anions involved the expansion of the unit-cell dimensions occurs in quite an usual range, but the *c*-axes become significantly longer than the *a*-axes (*a*-axes: from 405.3 pm to 408.6 pm; *c*-axes: from 679.9 pm to 916.2 pm) along the Cl^-^–Br^-^–I^-^ track. The lattice parameters of single crystalline PrOBr (*a* = 406.71 pm, *c* = 746.69 pm) fit almost perfectly with that from a previous powder diffraction study (*a* = 407.1 pm, *c* = 748.7 pm; Mayer *et al.* 1965). Differences in the molar volumes of the PbFCl-type praseodymium(III) oxide halides (*V_m_*(PrOCl) = 33.6 cm^3^/mol, *V_m_*(PrOBr) = 37.2 cm^3^/mol, *V_m_*(PrOI) = 46.1 cm^3^/mol) correspond well with the differences of the molar volumes of the respective halide anions (*V_m_*(Cl^-^) = 16.3 cm^3^/mol, *V_m_*(Br^-^) = 19.2 cm^3^/mol, *V_m_*(I^-^) = 24.5 cm^3^/mol; Biltz 1934). However, the Pr^3+^ cations occupy the *Wyckoff* site 2*c* (symmetry: 4*mm*) and bond four O^2-^ anions as well as four+*one*+*one* Br^-^ anions ending up with a total coordination number of 8+*1*+*1* (Figure 1). While the Br^-^ anions exhibit the same site symmetry as the Pr^3+^ cations, the oxide anions are located at *Wyckoff* position 2*a* with the site symmetry 4*m*2. *Bond-Valence* and *MAPLE* calculations support the interpretation of one important (*d*(Pr^3+^···Br^-^) = 360.8 (1) pm) and one less important secondary contact (*d*(Pr^3+^···Br^-^) = 385.9 (1) pm): The valency and ECoN for the first bond amounts to values of about 0.08 (with *R*_0_ = 267 pm, *b* = 37 pm; Brown, 2002) and 0.12 (Hoppe, 1975), but almost *nil* for the second one, since this next nearest contact to bromide has only very low influence on the effective coordination sphere of the Pr^3+^ cations (ECoN = 0.03).

### Experimental

Pale green, transparent, plate-shaped single crystals of PrOBr were obtained as by-product from a mixture of 0.06 g Pr, 0.38 g PrBr_3_ and 0.01 g NaN_3_, along with 0.30 g NaBr added as a flux. The mixture was kept at 800 °C for 7 days in an evacuated, sealed fused-silica vessel designed to produce the praseodymium(III) nitride bromide Pr_3_NBr_6 _in analogy with La_3_NBr_6_ (Lulei, 1998) and Ce_3_NBr_6_ (Mattausch & Simon, 1996).

### Refinement

The highest peak and the deepest hole in the final difference Fourier map are 95 pm and 84 pm apart from Pr.

### Crystal data

**Table:**

PrBrO	*D*_x_ = 6.368 Mg m^−^^3^
*M**_r_* = 236.82	Mo *K*α radiation, λ = 0.71069 Å
Tetragonal, *P*4/*n**m**m*	Cell parameters from 3957 reflections
Hall symbol: -P 4a 2a	θ = 0.4–27.9°
*a* = 4.0671 (3) Å	µ = 35.52 mm^−^^1^
*c* = 7.4669 (5) Å	*T* = 293 K
*V* = 123.51 (2) Å^3^	Plate, pale green
*Z* = 2	0.11 × 0.07 × 0.02 mm
*F*(000) = 204	

### Data collection

**Table:**

Bruker–Nonius KappaCCD diffractometer	113 independent reflections
Radiation source: fine-focus sealed tube	111 reflections with *I* > 2σ(*I*)
graphite	*R*_int_ = 0.082
ω and φ scans	θ_max_ = 27.9°, θ_min_ = 5.5°
Absorption correction: numerical (*X-SHAPE*; Stoe & Cie, 1999)	*h* = −5→5
*T*_min_ = 0.049, *T*_max_ = 0.535	*k* = −5→5
1621 measured reflections	*l* = −9→9

### Refinement

**Table:**

Refinement on *F*^2^	Primary atom site location: structure-invariant direct methods
Least-squares matrix: full	Secondary atom site location: difference Fourier map
*R*[*F*^2^ > 2σ(*F*^2^)] = 0.026	*w* = 1/[σ^2^(*F*_o_^2^) + (0.0378*P*)^2^] where *P* = (*F*_o_^2^ + 2*F*_c_^2^)/3
*wR*(*F*^2^) = 0.059	(Δ/σ)_max_ < 0.001
*S* = 1.20	Δρ_max_ = 1.14 e Å^−^^3^
113 reflections	Δρ_min_ = −2.52 e Å^−^^3^
10 parameters	Extinction correction: *SHELXL97* (Sheldrick, 2008), Fc^*^=kFc[1+0.001xFc^2^λ^3^/sin(2θ)]^-1/4^
0 restraints	Extinction coefficient: 0.032 (5)

### Special details

**Table:**

Geometry. All esds (except the esd in the dihedral angle between two l.s. planes) are estimated using the full covariance matrix. The cell esds are taken into account individually in the estimation of esds in distances, angles and torsion angles; correlations between esds in cell parameters are only used when they are defined by crystal symmetry. An approximate (isotropic) treatment of cell esds is used for estimating esds involving l.s. planes.
Refinement. Refinement of F^2^ against ALL reflections. The weighted R-factor wR and goodness of fit S are based on F^2^, conventional R-factors R are based on F, with F set to zero for negative F^2^. The threshold expression of F^2^ > 2sigma(F^2^) is used only for calculating R-factors(gt) etc. and is not relevant to the choice of reflections for refinement. R-factors based on F^2^ are statistically about twice as large as those based on F, and R- factors based on ALL data will be even larger.

### Fractional atomic coordinates and isotropic or equivalent isotropic displacement parameters (Å^2^)

**Table:**

	*x*	*y*	*z*	*U*_iso_*/*U*_eq_	
Pr	0.2500	0.2500	0.15763 (8)	0.0106 (4)	
O	0.7500	0.2500	0.0000	0.0129 (13)	
Br	0.2500	0.2500	0.64087 (17)	0.0153 (4)	

### Atomic displacement parameters (Å^2^)

**Table:**

	*U*^11^	*U*^22^	*U*^33^	*U*^12^	*U*^13^	*U*^23^
Pr	0.0084 (4)	0.0084 (4)	0.0148 (6)	0.000	0.000	0.000
O	0.0114 (17)	0.0114 (17)	0.016 (3)	0.000	0.000	0.000
Br	0.0149 (5)	0.0149 (5)	0.0160 (7)	0.000	0.000	0.000

### Geometric parameters (Å, °)

**Table:**

Pr—O^i^	2.3496 (3)	Pr—Pr^i^	3.7165 (8)
Pr—O^ii^	2.3496 (3)	Pr—Pr^x^	3.7165 (8)
Pr—O^iii^	2.3496 (3)	Pr—Pr^iii^	3.7165 (8)
Pr—O	2.3496 (3)	O—Pr^i^	2.3496 (3)
Pr—Br^iv^	3.2457 (8)	O—Pr^xi^	2.3496 (3)
Pr—Br^v^	3.2457 (8)	O—Pr^iii^	2.3496 (3)
Pr—Br^vi^	3.2457 (8)	Br—Pr^iv^	3.2457 (8)
Pr—Br^vii^	3.2457 (8)	Br—Pr^v^	3.2457 (8)
Pr—Br	3.6083 (14)	Br—Pr^vii^	3.2457 (8)
Pr—Br^viii^	3.8586 (14)	Br—Pr^vi^	3.2457 (8)
Pr—Pr^ix^	3.7165 (8)		
			
O^i^—Pr—O^ii^	75.466 (11)	O—Pr—Pr^i^	37.733 (6)
O^i^—Pr—O^iii^	119.87 (3)	Br^iv^—Pr—Pr^i^	107.075 (12)
O^ii^—Pr—O^iii^	75.466 (11)	Br^v^—Pr—Pr^i^	107.075 (12)
O^i^—Pr—O	75.466 (11)	Br^vi^—Pr—Pr^i^	168.31 (4)
O^ii^—Pr—O	119.87 (3)	Br^vii^—Pr—Pr^i^	66.920 (18)
O^iii^—Pr—O	75.466 (11)	Pr^ix^—Pr—Pr^i^	66.346 (15)
O^i^—Pr—Br^iv^	140.758 (5)	O^i^—Pr—Pr^x^	98.99 (2)
O^ii^—Pr—Br^iv^	140.758 (5)	O^ii^—Pr—Pr^x^	37.733 (6)
O^iii^—Pr—Br^iv^	71.938 (15)	O^iii^—Pr—Pr^x^	37.733 (6)
O—Pr—Br^iv^	71.938 (15)	O—Pr—Pr^x^	98.99 (2)
O^i^—Pr—Br^v^	71.938 (15)	Br^iv^—Pr—Pr^x^	107.075 (12)
O^ii^—Pr—Br^v^	71.938 (15)	Br^v^—Pr—Pr^x^	107.075 (12)
O^iii^—Pr—Br^v^	140.758 (5)	Br^vi^—Pr—Pr^x^	66.920 (18)
O—Pr—Br^v^	140.758 (5)	Br^vii^—Pr—Pr^x^	168.31 (4)
Br^iv^—Pr—Br^v^	124.77 (5)	Pr^ix^—Pr—Pr^x^	66.346 (15)
O^i^—Pr—Br^vi^	140.758 (5)	Pr^i^—Pr—Pr^x^	101.39 (3)
O^ii^—Pr—Br^vi^	71.938 (15)	O^i^—Pr—Pr^iii^	98.99 (2)
O^iii^—Pr—Br^vi^	71.938 (15)	O^ii^—Pr—Pr^iii^	98.99 (2)
O—Pr—Br^vi^	140.758 (5)	O^iii^—Pr—Pr^iii^	37.733 (6)
Br^iv^—Pr—Br^vi^	77.59 (2)	O—Pr—Pr^iii^	37.733 (6)
Br^v^—Pr—Br^vi^	77.59 (2)	Br^iv^—Pr—Pr^iii^	66.920 (19)
O^i^—Pr—Br^vii^	71.938 (15)	Br^v^—Pr—Pr^iii^	168.31 (4)
O^ii^—Pr—Br^vii^	140.758 (5)	Br^vi^—Pr—Pr^iii^	107.075 (12)
O^iii^—Pr—Br^vii^	140.758 (6)	Br^vii^—Pr—Pr^iii^	107.075 (12)
O—Pr—Br^vii^	71.938 (15)	Pr^ix^—Pr—Pr^iii^	101.39 (3)
Br^iv^—Pr—Br^vii^	77.59 (2)	Pr^i^—Pr—Pr^iii^	66.346 (15)
Br^v^—Pr—Br^vii^	77.59 (2)	Pr^x^—Pr—Pr^iii^	66.346 (15)
Br^vi^—Pr—Br^vii^	124.77 (5)	Pr—O—Pr^i^	104.534 (11)
O^i^—Pr—Pr^ix^	37.733 (6)	Pr—O—Pr^xi^	119.87 (3)
O^ii^—Pr—Pr^ix^	37.733 (6)	Pr^i^—O—Pr^xi^	104.534 (11)
O^iii^—Pr—Pr^ix^	98.99 (2)	Pr—O—Pr^iii^	104.534 (11)
O—Pr—Pr^ix^	98.99 (2)	Pr^i^—O—Pr^iii^	119.87 (3)
Br^iv^—Pr—Pr^ix^	168.31 (4)	Pr^xi^—O—Pr^iii^	104.534 (11)
Br^v^—Pr—Pr^ix^	66.920 (19)	Pr^iv^—Br—Pr^v^	124.77 (5)
Br^vi^—Pr—Pr^ix^	107.075 (12)	Pr^iv^—Br—Pr^vii^	77.59 (2)
Br^vii^—Pr—Pr^ix^	107.075 (12)	Pr^v^—Br—Pr^vii^	77.59 (2)
O^i^—Pr—Pr^i^	37.733 (6)	Pr^iv^—Br—Pr^vi^	77.59 (2)
O^ii^—Pr—Pr^i^	98.99 (2)	Pr^v^—Br—Pr^vi^	77.59 (2)
O^iii^—Pr—Pr^i^	98.99 (2)	Pr^vii^—Br—Pr^vi^	124.77 (5)

Symmetry codes: (i) −*x*+1, −*y*, −*z*; (ii) *x*−1, *y*, *z*; (iii) −*x*+1, −*y*+1, −*z*; (iv) −*x*+1, −*y*+1, −*z*+1; (v) −*x*, −*y*, −*z*+1; (vi) −*x*, −*y*+1, −*z*+1; (vii) −*x*+1, −*y*, −*z*+1; (viii) *x*, *y*, *z*−1; (ix) −*x*, −*y*, −*z*; (x) −*x*, −*y*+1, −*z*; (xi) *x*+1, *y*, *z*.
